# Functional Modulation of Gut Microbiota and Blood Parameters in Diabetic Rats Following Dietary Intervention with Free or Immobilized *Pediococcus acidilactici* SK Cells on Pistachio Nuts

**DOI:** 10.3390/nu16234221

**Published:** 2024-12-06

**Authors:** Ioanna Prapa, Amalia E. Yanni, Vasiliki Kompoura, Gregoria Mitropoulou, Panayiotis Panas, Nikolaos Kostomitsopoulos, Yiannis Kourkoutas

**Affiliations:** 1 Laboratory of Applied Microbiology and Biotechnology, Department of Molecular Biology and Genetics, Democritus University of Thrace, 68100 Alexandroupolis, Greece; ioannaprap@gmail.com (I.P.); vickykom20.70@gmail.com (V.K.); grigoriamitropoulou@gmail.com (G.M.); 2 Laboratory of Chemistry, Biochemistry, Physical Chemistry of Foods, Department of Nutrition and Dietetics, Harokopio University of Athens, 17671 Athens, Greece; 3 QLC, N.E.O. Patron-Athinon 57, 26442 Patras, Greece; panas@qlc.gr; 4 Laboratory Animal Facility, Biomedical Research Foundation of the Academy of Athens, 11527 Athens, Greece; nkostom@bioacademy.gr

**Keywords:** cell immobilization, gut microbiome, HDL-c, IL-1β, *Pediococcus acidilactici*, pistachio, probiotics, type 1 diabetes mellitus

## Abstract

Background/Objectives: The gut microbiota is linked to the pathogenesis of type 1 diabetes mellitus (T1DM), while supplementation with probiotics may result in positive alterations in the composition of the gut microbiome. This research aimed to map the changes in the gut microbiome and blood markers of streptozotocin-induced diabetic rats after a dietary intervention with free or immobilized cells of the presumptive probiotic *Pediococcus acidilactici* SK on pistachio nuts. Methods: Twenty-four male Wistar rats were studied and divided into four groups (healthy or diabetic) which received the free or the immobilized *P*. *acidilactici* SK cells on pistachio nuts for 4 weeks. Blood, fecal, and intestinal tissue samples were examined. Results: The diabetic rats exhibited an elevated concentration of HDL-c, while the inflammatory IL-1β levels were significantly lower in the diabetic animals that received the immobilized cells compared to the group that received the free cells. The dietary intervention with immobilized cells led to decreased counts of fecal staphylococci and enterococci in the diabetic animals, while the diet with both free and immobilized *P*. *acidilactici* SK cells rendered levels of these populations in normal values in the feces and intestinal tissue of the diabetic animals. Noticeably, the *Lactobacillus* and *Bifidobacterium* genera were elevated after the supplementation with immobilized *P. acidilactici* SK cells on pistachio nuts. Conclusions: Dietary supplementation with *P. acidilactici* SK cells (in free or in immobilized form) beneficially affected the gut microbiota/microbiome of streptozotocin-induced diabetic rats, leading to the alleviation of dysbiosis and inflammation and control over their lipid levels.

## 1. Introduction

According to the latest data, in 2022, there were about 8.75 million people worldwide suffering from type 1 diabetes mellitus (T1DM) [1], while it is projected that this number could be nearly duplicated and rise to 13.5–17.4 million by 2040 [2]. The onset of T1DM is characterized by a humoral and cellular autoimmune response towards the β-cells of the Langerhans islets [3]. Multiple synergistic effects of a person’s genetic, immunological, and environmental background can result in the multifactorial disorder of T1DM [4] and pancreatic cell autoimmunity-driven destruction [5].

Dysbiosis, increased intestinal permeability, and induced systemic inflammation together with exposure to microbial antigens have been associated with T1DM progression [6], whereas the exact mechanisms that lead to autoimmunity (and thus to T1DM) are not fully understood. Probiotic usage has been linked to reduced β-cell autoimmunity in T1DM-prone children [7].

Probiotic microorganisms [8] affect the composition of the intestinal microbiota when consumed in adequate amounts and may confer health benefits to the host. In T1DM, the consumption of probiotics can induce a better glycemic response [9] and decrease the level of pro-inflammatory cytokines (IL-1β, IL-6, and TNF-a), while increasing that of anti-inflammatory cytokines (TGF-β and IL-10) [10]. Poor glycemic control has been associated with dyslipidemia, i.e., elevated cholesterol levels, reduced HDL-c levels, and increased LDL-c levels in T1DM [11]. Management of the lipid profile in T1DM is of great importance, as it reduces the risk of cardiovascular complications [11]. *Lactobacillus johnsonii* supplementation delayed or inhibited the onset of T1DM in rat animal models [12], while *Bifidobacterium lactis* improved the insulin tolerance and fatty acid profiles in diabetic mice [13].

The composition of the intestinal microbiota is influenced by many factors, including a person’s mode of delivery, breastfeeding status, age, diseases (presence of any autoimmune or chronic disease) or allergies, mental health (depression, stress, mood disorders), physical activity, antibiotic usage, smoking, alcohol drinking, and dietary habits [14]. Dietary patterns (e.g., Mediterranean diet) and the variety of dietary ingredients shape the microbiome and alter the metabolic function of host cells [15]. Pistachio nuts (*Pistacia vera* L.) are widely consumed globally, and their health benefits have been widely discussed [16]. This nut is rich in monounsaturated fats (mainly oleic acid), dietary fiber, protein, micronutrients, and tocopherols, while it contains a low amount of saturated fats [16]. Recent reports [17,18] underline that the functional phytochemicals and polyphenols of pistachios remain bio-accessible after their in vitro gastrointestinal (GI) digestion; therefore, these compounds can reach the large intestine, be available as prebiotic fibers, and induce alterations in the microbiota. Furthermore, the effects of pistachio nut consumption on the intestinal microbiome of T1DM animal models [19], high-fat-diet (HFD)-fed rats [20], healthy volunteers [21], and patients with diabetes (Type-2 Diabetes Mellitus) [22,23] have been explored.

The immobilization of probiotics on natural food ingredients is a naturally occurring process that, when combined with a drying method (such as freeze-drying), can lead to the development of functional food constituents [24,25]. It has been well documented that the viability of freeze-dried immobilized cells is significantly increased during storage [25] and during digestion [26] compared to free cells. Substrates containing prebiotic fibers can be used as immobilization vehicles and thus allow the production of synbiotic food constituents that could display functional properties and enhanced potential health benefits [27] in metabolic diseases like T1DM.

Presumptive probiotic cells of *Pediococcus acidilactici* SK, isolated from human stool, have shown promising probiotic properties after their in vitro evaluation and assessment of their safety [28]. What is more, after an in vivo dietary intervention in HFD-fed mice, significantly improved insulin resistance was noticed in HFD-fed mice that received *P. acidilactici* SK cells compared to a standard diet-fed group [28]. This strain belongs to the *P. acidilactici* genus, which is included in the qualified presumption of safety (QPS) list of microorganisms that are recommended to be intentionally added to food or feed, as notified by EFSA [29].

The aim of the present study was to investigate the potential beneficial effects of a dietary intervention with free or immobilized *Pediococcus acidilactici* SK cells on pistachio nuts in a streptozotocin (STZ)-induced animal model of T1DM. The rats’ blood biochemical parameters, inflammatory factors, intestinal microbiome composition, and stool fatty acid profiles were examined.

## 2. Materials and Methods

### 2.1. Microbial Strains

*Pediococcus acidilactici* SK, isolated from human stool, kindly provided from QLC (26442, Patras, Greece), was used in the present study and was chosen due to its potential probiotic properties, as shown after in vitro and in vivo evaluation [28]. Cells were kept in −80 °C stock culture vials and retrieved in 10 mL of De Man–Rogosa–Sharpe (MRS) broth (VWR International, Radnor, PA, USA) followed by overnight incubation at 37 °C. A subculture was prepared in 10 mL MRS broth and incubated at 37 °C for 24 h prior to use.

### 2.2. Immobilization of Cells on Pistachio Nuts and Freeze-Drying

Cell immobilization on pistachio nuts (*Pistacia vera* L.) was performed as described recently [25]. In brief, pistachio nuts (kindly provided by Agricultural Pistachios Cooperation of Molos-Thermopyles, Greece) were introduced in fully grown cell suspension of *P. acidilactici* SK in sterile ¼ Ringer’s (VWR International) solution. After 24 h incubation of the food ingredient in the cell suspension, the mixture was strained and immobilized cells were washed with ¼ Ringer’s solution to remove non-immobilized cells. For comparison purposes, cell biomass (non-immobilized cells) was also prepared after centrifugation of grown culture on MRS broth, followed by a washing step with sterile ¼ Ringer’s solution. The cell pellet and the immobilized cells on pistachios were transferred to −80 °C for 18 h, followed by freeze-drying on a BenchTop Pro (Virtis, SP Scientific, Warminster, PA, USA) freeze-dryer under 30–35 Pa and condenser temperature of −101 °C for 24 h. Cell levels of freeze-dried free or immobilized *P. acidilactici* SK cells were determined after 10-fold serial dilutions and plating on MRS agar (Condalab, Madrid, Spain) followed by incubation at 37 °C for 72 h in anaerobic conditions (Anaerobic Jar 2.5 L & Anaerocult A, Merck Millipore, Merck KGaA, Darmstadt, Germany), as described below.

### 2.3. Scanning Electron Microscopy

The immobilization of *P. acidilactici* SK cells on pistachio nuts was verified via scanning electron microscopy [26]. A BalTec MED 020 Sputter coating system (Zürich, Switzerland) was used to coat freeze-dried immobilized cells with gold over a 2 min period, and cells were then further observed with a JSM-6300 scanning electron microscope (JEOL Ltd., Tokyo, Japan) fixed at 20 kV voltage.

### 2.4. In Vivo Study Design

Fourteen-week-old male RccHan^®^: WIST rats (350–400 g body weight) were randomly divided into 4 groups (*n* = 6 per group). The minimum number of animals that were needed to observe significant differences was calculated after power analysis using the “G*Power v. 3.1.9.4” software. F-test analysis of variance (ANOVA) was conducted using data from a previous pilot study [19]. Given the losses observed after STZ injection (~1/4), 6 animals per group were used, a sufficient number to draw significant results, as shown in previous studies [19,30]. During the experimental procedure, all rats were individually housed in polysulfone cages (Blue Line, Tecniplast, Buguggiate, Italy) in the Laboratory Animal Facility of the Biomedical Research Foundation of the Academy of Athens (BRFAA), Greece. Controlled temperature (21 ± 2 °C), relative humidity (55 ± 10%), and a 12 h light/dark cycle (light period between 7:00 and 19:00) were applied, and the well-being of the animal models was monitored by the specialized staff of the facility. The cage bedding comprised corncob granules (REHOFIX^®^, J. Rettenmaier & Söhne GmbH + Co KG, Rosenberg, Germany). Cages and bedding were changed every four days. The research protocol was conducted according to the European Directive 2010/63 and was approved by the Veterinary Directorate of the Athens Prefecture (reference number 2057/05-04-2017) and by the Committees on Research Ethics of Democritus University of Thrace (reference number 9254/386/22-05-2020) and οf BRFAA (reference number 90/19-05-2021).

#### 2.4.1. STZ-Induced Animal Model

T1DM was induced using STZ, as described previously [30]. In brief, animals in non-fasting state received an intraperitoneal injection of STZ (Sigma-Aldrich, Merck KGaA, Darmstadt, Germany) in citrate buffer (0.1 M, pH 4.5) solution in a dosage of 60 mg/kg of body weight [30]. After 1 week, animals with blood glucose levels ≥ 250 mg/dL were considered diabetic and included in this study. T1DM was accompanied by signs of polyuria and polydipsia [31]. Body weight and glucose levels were determined every week to assure the establishment and maintenance of T1DM; for the glucose measurements, a digital glucose meter (OneTouch Verio FlexTM, Lifescan Canada Ltd., Burnaby, BC, Canada) was used.

#### 2.4.2. Dietary Intervention with Probiotic Strain

**Figure 1:**
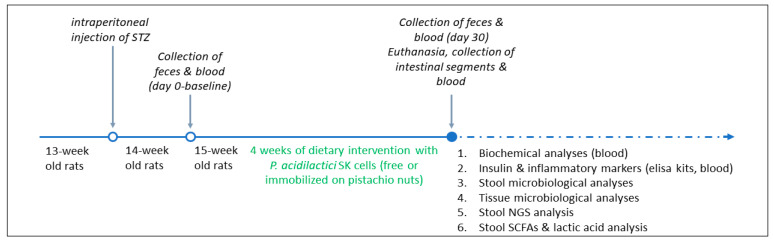
Schematic representation of in vivo experimental design.

Animal models were divided into 4 groups based on their dietary treatment as follows: healthy animals that received the immobilized *P. acidilactici* SK cells on pistachio nuts (*n* = 6, HIP) or the free *P. acidilactici* SK cells (*n* = 6, HFP) and STZ-induced diabetic animals that received the immobilized *P. acidilactici* SK cells on pistachio nuts (*n* = 6, DIP) or the free *P. acidilactici* SK cells (*n* = 6, DFP) (Figure 1). Based on previous studies exploring the effects of pistachio nut supplementation on STZ-induced diabetic rats [19,32], the dietary intervention lasted for 4 weeks. The daily dose was 2 × 10^9^ cfu of probiotic cells [33]. This amount of cells was achieved by administrating 2 g of pistachio nuts (corresponding to 2–3 pistachios) to each animal daily. Nutrient composition and energy content (approximately 430 kcal/100 g) of the different dietary regimens administrated to rats in this study have been presented previously [19,32]. In control groups receiving free (non-immobilized) cells, rat chow was enriched with corn oil to match the fat content (10% *w*/*w*) of the pistachio nuts diet [19,32]. To ensure consistent probiotic intake, the proper amount of food was administered daily to ensure that each animal consumed the required cell dose (in most cases no remains were left).

### 2.5. Sample Collection

Heparinized plasma samples (from the lateral tail vein) were collected at baseline (day 0, week 0), while animals were in a fasting state, and at the end of this study (day 30, week 4) and were stored at −80 °C, until analysis of biochemical parameters and insulin determination.

Fecal samples were collected at baseline and at the end (4th week) of the dietary intervention and stored in sterile tubes at −80 °C. After 4 weeks of the dietary intervention, the animals were anesthetized by inhalation of isoflurane (ISOVET, Chanelle Pharma, Loughrea, Co., Ltd., Galway, Ireland), followed by euthanasia by exsanguination after the collection of blood from the anterior vena cava. Small and large intestinal segments of jejunum, ileum, cecum, and colon were aseptically dissected and rinsed with sterile saline solution twice in order to remove all intestinal fluid content. Immediately, tissue segment samples were diluted in 25% glycerol–Ringer’s solution (1:1) and stored in sterile tubes at −80 °C, until analysis.

Plasma was isolated from the blood samples and stored at −80 °C until analysis of inflammatory factors.

### 2.6. Sample Analysis

#### 2.6.1. Blood Analyses

Plasma glucose, total cholesterol (TC), high density lipoproteins’ cholesterol (HDL-c), low density lipoproteins’ cholesterol (LDL-c), and triacylglycerols (TAG) were determined on an automated biochemical analyzer (Konelab 60i, Thermo Fisher Scientific Inc., Waltham, MA, USA) using commercially available kits (Thermo Fisher Scientific Inc.). Plasma insulin levels at the beginning and the end of the dietary intervention were measured by a sandwich ELISA method using a commercially available rat insulin ELISA kit (EZRMI-13K, Merck Millipore). Inflammatory factors IL-1β, IL-6, and TNF-a were determined in plasma samples obtained from cardiac puncture at the end of the intervention using commercially available ELISA kits (OriGene, Rockville, MD, USA).

#### 2.6.2. Stool and Tissue Microbiota Analyses

To determine microbial populations, stool (1–2 g) or tissue samples (0.5–2 g) were homogenized in 0.1% buffered peptone water (Condalab), followed by serial decimal dilutions in ¼ Ringer’s solution, and checked for: (1) total aerobic counts (TAC) on plate count agar (Condalab) at 30 °C for 72 h; (2) coliforms on Violet Red Bile agar (Condalab) at 37 °C for 24 h; (3) *Enterobacteriacae* on Violet Red Bile Glucose agar (Condalab) at 37 °C for 24 h; (3) staphylococci in Baird Parker (Condalab) enriched with egg yolk tellurite (Condalab) at 37 °C for 48 h; (4) *Escherichia coli* in Harlequin TBGA/TBX (Condalab) at 37 °C for 24 h; (5) streptococci (white colonies) and entetococci (black colonies) in Kanamycin Aesculin Azide agar (Condalab) at 37 °C for 48 h; (6) lactic acid bacteria (LAB) in acidified MRS agar (Condalab) at 37 °C for 72 h anaerobically (Anaerobic Jar 2.5 L & Anaerocult A, Merck Millipore); and (7) bifidobacteria in Bifidobacteria agar (22 g/L bacteriological peptone, 5 g/L NaCl, 5 g/L dextrose, 1 g/L starch, 0.3 g/L L-cysteine HCl, 15 g/L agar) at 37 °C for 48 h anaerobically (Anaerobic Jar 2.5 L & Anaerocult A, Merck Millipore).

Incubation time was extended up to 120 h, until no extra colonies were observed. Plates containing 30–300 colonies were counted and results were expressed as log of mean colony forming units (cfu) per gram of fecal samples.

#### 2.6.3. DNA Extraction, PCR Amplification and 16S rRNA Sequencing

Total DNA isolation using NucleoSpin Stool Mini Kit (Macherey-Nagel, Duren, Germany), following manufacturer’s instructions, was performed in duplicate in fecal samples for days 0 and 30 for the 4 dietary groups (HIP, DIP, HFP, DFP).

Next-generation sequencing (NGS) was performed using MiSeq sequencing by MR DNA (www.mrdnalab.com, Shallowater, TX, USA), as previously described [19,30]. To evaluate the microbiome compositions of the samples, universal prokaryotic primers 27F (AGRGTTTATCMTGGCTCAG) and 519R (GTNTTACNGCGGCKGCTG), targeting the V1–V3 hypervariable regions of the 16S rRNA gene [34], were utilized on the Illumina MiSeq 2 × 300 PE sequencing platform (Illumina, San Diego, CA, USA). PCR conditions and product purification were set as described recently [30]. Then, using MiSeq, sequencing samples were prepared for the illumina DNA library, following the manufacturer’s instructions. Sequencing data processing was accomplished using an in-house analysis pipeline by MR DNA. Operational taxonomic units (OTUs) were defined by clustering at 3% divergence (97% similarity) and the final OTUs were taxonomically classified using BLASTn against a curated database derived from RDPII and NCBI (www.ncbi.nlm.nih.gov). Subsequent analysis of raw data and calculation of a-diversity were performed using R Studio v. 2023.09.1+494 [35] and Rhea platform scripts [36].

#### 2.6.4. Stool Short Chain Fatty Acids (SCFAs) and Lactic Acid Profile

Fatty acid purification and extraction from feces was performed, as described previously [32]. SCFAs (acetic, propionic, butyric, isobutyric, valeric, and isovaleric acids), as well as lactic acid concentrations, were determined by HPLC, using a Shimadzu chromatography system (Shimadzu Corp., Duisburg, Germany). Fecal SCFAs and lactate concentrations were expressed as mean μmol per gram of feces, using the following equation [37]:

SCFAs/lactate (μmol/g) = [organic acid in fecal contents (mmol/mL) × Vd (mL) × 1000]/weight of feces (g)

where: Vd = Volume of Dilution.

### 2.7. Statistical Analysis

Data are expressed as the mean values ± SEM (standard error of the mean). Statistical software Statistica v. 12 (StatSoft, Inc., Tulsa, OK, USA) was used to perform the analysis. ANOVA was applied to assess the effect of factors on the variables. Specifically, one-way ANOVA coupled with the Bonferroni post-hoc test was used to compare microbiota populations in intestinal segments and inflammatory markers in the four groups of animals, while two-way ANOVA followed by the Bonferroni post-hoc test was applied to compare microbiota populations in feces, as well as, for the body weight, blood analyses (biochemical and insulin), and SCFAs, in the four groups of animals and at the 2 timepoints (baseline and 4th week). For all statistical analyses, statistical significance was set at *p* < 0.05.

## 3. Results and Discussion

Functional foods containing health-promoting probiotic microorganisms have been shown to act synergistically with insulin, stabilizing the glucose levels of patients with T1DM [38] or by ameliorating the abnormal lipid profile [39]. In general, the consumption of probiotics potentially affects the microbial ecology of the gut in a beneficial way [40,41]. Lower insulin requirements and improved levels of HbA1c were observed, suggesting a supportive role of probiotics in controlling glycemia in children with newly diagnosed T1DM [9], while another clinical trial highlighted alterations in inflammatory mediators after the consumption of probiotics [6].

In the present study, the presumptive probiotic *P. acidilactici* SK strain that was isolated from human stool samples was immobilized on pistachio nuts [25], given their potential to modulate the intestinal microbiome [19] and fatty acid profiles [32] of diabetic rats. The strain was chosen due to its potential probiotic properties, according to in vitro data and the previous finding that its supplementation in diet-induced obese mice resulted in a delayed development of insulin resistance [28]. Functional food constituents containing 2 × 10^9^ cells were administered daily to STZ-induced diabetic rats for 4 weeks and differences in the microbiota/microbiome composition of the feces and intestinal tissue along with biochemical parameters, inflammatory markers, and the fecal SCFA profiles of the rats were investigated.

### 3.1. Immobilized P. acidilactici SK Cells on Pistachio Nuts

**Figure 2:**
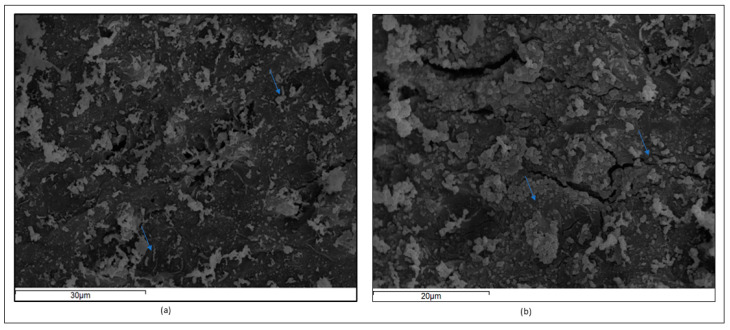
Electron micrographs of immobilized *P. acidilactici* SK cells on pistachio nuts at (**a**) 30 μm and (**b**) 20 μm scale. Arrows indicate the cell aggregates.

Cell immobilization technology is proposed as a useful tool to achieve high cell loads of probiotic cultures, along with increased viability during their digestion [26] and storage [25], which are both crucial factors in the development of functional food ingredients [42]. The use of immobilized probiotics on natural food ingredients could proffer synergistic health benefits by combining the nutritional properties of the food ingredient (e.g., pistachio nuts) with the presumptive beneficial effects of probiotics. The immobilization and freeze-drying of *P. acidilactici* SK cells on pistachio nuts, which have been shown to exert prebiotic-like properties, as they can shape the intestinal microbiota [19], resulted in concentrations of 1.3 × 10^9^ cfu/g that remained stable after storage at 4 °C for 4 weeks [25]. Representative micrographs of immobilized cells on pistachio nuts after SEM microscopy are shown in Figure 2. Cell aggregates which are formed after the immobilization on pistachio nuts are visible in the photographs, indicating a “cell-mating”, which was also reported in similar studies [26,43]. Notably, through the administration of a 10% *w*/*w*-enriched diet with pistachio nuts, a dosage of 2 × 10^9^ cfu/day was achieved.

### 3.2. Body Weight, Biochemical Profile, Insulin, and Inflammatory Factors

Both the dietary intervention (*p* = 0.001) and the time (*p* = 0.022) had a significant effect on the rats’ body weight and a strong (*p* = 0.001) interaction between the two factors was also observed. The body weight of the animal models was not affected by the probiotic dietary intervention that lasted for 4 weeks (Table 1). The STZ-induced diabetic animals (groups DIP and DFP) showed a significant (*p* = 0.001 for DIP and *p* = 0.007 for DFP, compared to baseline values) and progressive loss of body weight after 4 weeks, which has also been reported elsewhere [30,44]. The progressive loss of body weight is an indicator/symptom of T1DM. The decreased body weight in the diabetic rats can be attributed to protein and fat catabolism caused by insulin deficiency [45]. However, supplementation with free or immobilized *P. acidilactici* SK cells could not counterpoise this effect.

Specific probiotics are known for their cholesterol-lowering properties, while their use in lipid control in T1DM is under investigation [46]. Probiotic consumption has been shown to regulate lipid [47] and glucose metabolism [48]. Novel wild-type strains that exert probiotic potential in vitro may possess similar functional properties in vivo [49].

Biochemical parameters of the four groups of animals at the beginning and the end of the study are presented in Table 1. ANOVA revealed that both the dietary intervention and the time had a significant effect on the animals’ glucose (*p* < 0.001 and *p* = 0.001, respectively) and TC (*p* = 0.002 and *p* = 0.040, respectively) levels, while the TAG (*p* = 0.026), LDL-c (*p* < 0.001), and insulin (*p* < 0.001) levels were only affected by the dietary intervention. Both factors had a significant (*p* < 0.05) effect on the HDL-c levels and a strong (*p* < 0.05) interaction was also observed. However, for TC, TAG, and LDL-c, Bonferroni post-hoc comparisons did not identify significant differences between the groups at the corresponding time points or within the same group over time.

The plasma glucose levels of the STZ-induced diabetic rats were significantly higher (*p* < 0.001) compared to the healthy groups (HIP and HFP) and ranged between 324–407 mg/dL. The glucose concentration was not affected by supplementation with immobilized or free *P. acidilactici* SK cells (*p* = 1.000). The plasma insulin levels were significantly lower in the diabetic rats compared to the healthy animals (*p* < 0.001 compared to HIP and HFP) and were also not affected by the dietary supplementation. It has been shown that the administration of 1.5 × 10^9^ cfu of *Lactobacillus acidophilus* FNCC 0051 for 21 days in STZ-induced diabetic rats resulted in a reduction in blood glucose levels, but did not reduce it to normal levels [50]. In C57BL/6L STZ-induced mice T1DM models, the consumption of *Levilactobacillus brevis* KLDS 1.0727 and *L. brevis* KLDS 1.0373 cells for 4 weeks resulted in reduced plasma glucose levels, due to the production of gamma-aminobutyric acid [51]. However, *L. brevis* is able to produce gamma-aminobutyric acid biologically as an intrinsic property [52], which is not the case for *P. acidilactici* cells. The use of probiotic-containing products by patients with T1DM has been reported to help maintain better glycemic control and ameliorate conditions of metabolic syndrome, such as high blood pressure, high TAG levels, and lower levels of HDL-c [53].

Regarding the lipid profile, the TC, TAG, and LDL-c levels remained within the normal range in the four groups of animals. At baseline, in the STZ-induced diabetic animals, lower (*p* < 0.001) levels of HDL-c compared to the healthy groups were noticed, in accordance with other studies [54,55]. After 4 weeks, the supplementation with both free or immobilized *P. acidilactici* SK cells led to increased levels of HDL-c (*p* = 0.043 and *p* = 0.014, for DIP and DFP, respectively, compared to baseline values) in the diabetic groups, but no difference between the DIP and DFP levels was observed. In the diabetic groups, the values for HDL-c were similar to those of the healthy groups (*p* = 1.000), which could be attributed to the dietary regimens (diet enriched with free or immobilized *P. acidilactici* SK cells on pistachio nuts), which could have affected the lipid profiles of diabetic rats. In T1DM, serum lipid abnormalities (dyslipidemia) occur, owing to the deficiency of insulin production [56]. Increased TAG and reduced HDL-c levels are the main results of this symptom [55]. Accordingly, in another study, the daily consumption of the *P. acidilactici* FZU106 strain, isolated from wine, in HFD-induced hyperlipidemic rats led to decreased TC, TAG, and LDL-c levels and increased HDL-c levels, along with beneficial alterations in the intestinal microbiome composition, after 8 weeks [46]. Other probiotic strains belonging to the *Lacticaseibacillus* genus (*L. paracasei* SD1 and *L. rhamnosus* SD1) and that were isolated from the oral microbiome of children improved the lipid profile of STZ- induced mice (T1DM); specifically, the probiotic consumption led to increased serum HDL-c levels after 4 weeks [45] along with the regulation of pancreatic inflammation and the modulation of glucotoxicity.

Probiotics can possess hypocholesterolemic effects that can be achieved through several proposed mechanisms: the deconjugation of bile salts, the regulation of lipid metabolism, the incorporation of cholesterol in the cell membrane of the probiotics, and the assimilation or conversion of cholesterol to coprostanol [57]. Lipoprotein (a) and low-density lipoproteins are highly atherogenic. HDL-c plays a pivotal role in metabolic diseases, due to the atheroprotective properties that they exhibit [58]. Numerous studies have shown that dyslipidemia is highly associated with T1DM, yet an increase in HDL-c levels could contribute positively to metabolic health in the condition of T1DM [59,60].

The inflammatory factors TNF-a and IL-1β are implicated in the disease of T1DM, exerting negative impacts on insulin secretion [61], while elevated IL-6 levels are a sign of diabetes risk, as this cytokine impairs β-cell function alone or in combination with IL-1β [62]. Increased levels of IL-1β have been reported in diabetic animal models [30,63] and in humans [64,65], in agreement with the results of the present study, where, in diabetic groups (DIP and DFP), the levels of IL-1β were significantly higher (*p* < 0.001) compared to those of healthy animals (HIP and HFP) (Table 2). What is more, in the DIP group, the levels of IL-1β were lower compared to the DFP group (*p* < 0.001). The dietary regimen with immobilized *P. acidilactici* SK cells on pistachio nuts could render lower inflammatory IL-1β levels than the dietary regimen with freshly incorporated *P. acidilactici* SK cells on rats’ corn-oil-enriched food. However, data from diabetic groups receiving no probiotics and solely pistachio nuts would be needed to better explain this effect.

In T1DM, as in other autoimmune diseases, a variety of systemic malfunctions which are related to immunological, metabolic, and gut microbiome pathways occur, leading to the diabetic state. The gut microbiome is characterized by plasticity, as it can adapt to numerous environmental shifts and has been reported to make a significant contribution to systemic inflammation [66]. Likewise, in some cases of autoimmune diseases, increased levels of certain cytokines (TNF-a, IL-6, and IL-17) have been associated with gut dysbiosis. The interplay between the immunological response and gut microbiota is of importance and could alleviate the symptoms of this autoimmune disease via targeted dietary shifts that could balance the microbial diversity or lower the presence of specific microbes linked to intestinal permeability and systemic inflammation [66]. Dolpady et al. [67] demonstrated that NOD mice (pre-diabetic mice model) that consumed probiotic VSL#3 were protected against T1DM. Specifically, their microbiota composition was altered, their IL-1β expression was inhibited, and their gut immunity was modulated in a beneficial way that reduced intestinal inflammation and restored gut immune homeostasis, providing a direct link between inflammation, autoimmunity, T1DM, and probiotics.

### 3.3. Fecal and Tissue Microbiota Analysis

ANOVA revealed that the TAC, *Enterobacteriaceae*, coliforms, and *E. coli* counts were significantly affected by the dietary intervention (*p* = 0.001, *p* < 0.001, *p* < 0.001, *p* < 0.001, respectively), whereas the enterococci counts were altered only by time (*p* < 0.001). On the other hand, both factors had a significant effect on the staphylococci (*p* = 0.002 and *p* < 0.001, respectively), streptococci (*p* < 0.001 and *p* = 0.020, respectively), LAB (*p* = 0.022 and *p* < 0.001, respectively), and bifidobacteria counts (*p* < 0.001 and *p* < 0.001, respectively). However, comparisons between groups and over time using Bonferroni post-hoc test pointed out specific significant differences, as indicated in Table 3. In detail, T1DM resulted in an elevated presence of *Enterobacteriaceae*, coliforms, and *E. coli* loads in stool samples at baseline (*p* < 0.001 for all populations). Likewise, the streptococci counts were higher in the diabetic animals at baseline compared to the healthy groups (*p* < 0.001, *p* = 0.046 between DIP and HIP, HFP and *p* = 0.003, *p* = 0.022 between DFP and HIP, HFP). Similar results were reported elsewhere [19,30]. *Enterobacteriaceae*, mainly comprised of opportunistic pathogens, have been found to be elevated in newly diagnosed T1DM-children [68]. These alterations can highlight the dysbiosis that is associated with T1DM.

The dietary intervention with immobilized *P. acidilactici* SK cells on pistachio nuts led to decreased levels of the commensal yet opportunistic pathogens staphylococci (*p* = 0.009) and enterococci (*p* = 0.028), while no such difference was observed (*p* = 0.058 and *p* = 0.818, respectively between baseline and week 4) in diabetic animals that received the free (non- immobilized) cell-enriched diet. After 4 weeks of the dietary intervention, the streptococci levels in both diabetic groups were similar to those of the healthy animals (*p* = 1.000). Of note, in diabetic rats that were administered the free *P. acidilactici* SK cells for 4 weeks, *Enterobacteriaceae*, coliforms, and *E. coli* were observed in similar levels (*p* = 1.000, *p* = 1.000 and *p* = 0.066, respectively, compared to HFP) to those of the healthy rats receiving the free cells; however, they were not significantly different compared to baseline values of the same group (*p* = 1.000 for all groups). Concerning the commensal LAB, after 4 weeks, the cell loads were increased in all groups compared to baseline values (*p* < 0.001 for all groups), indicating the survival of the administrated strain that belongs to LAB. The bifidobacteria counts were significantly increased (*p* = 0.002) in healthy rats receiving the immobilized cells on pistachio nuts, compared to their initial cell loads. Such differences can be perceived as beneficial alterations in the microbiota and could be attributed to the dietary intervention with the presumptive probiotic cells. Probiotics can restore the fecal microbiota balance after enhancing the growth of beneficial populations, along with limiting the presence of harmful bacteria [69]. The exact mechanisms by which this is accomplished are not fully understood, but the proposed ways include: (a) the inhibition of pathogens owing to the production of antimicrobial substances (SCFAs, peptides or toxins) [70]; (b) antagonistic action towards the colonization of the intestinal epithelium [71]. No significant differences (*p* > 0.05) were observed between the fecal microbiota populations of the DIP and DFP groups.

**Figure 3:**
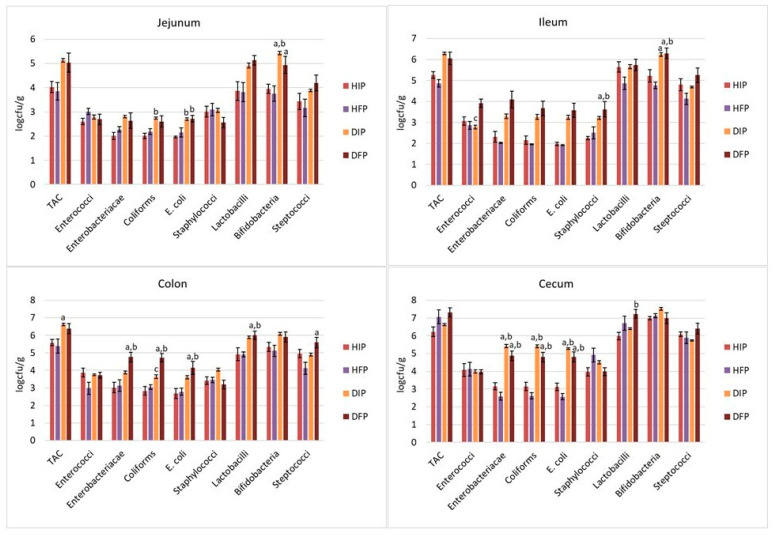
Effect of dietary intervention with free or immobilized *P. acidilactici* SK cells on pistachio nuts on intestinal microbiota populations of the four different intestinal segments (jejunum, ileum, cecum, and colon) in healthy and diabetic animals. Values are expressed as mean ± SEM (*n* = 6 per group). HIP: healthy animals that received the immobilized *P. acidilactici* SK cells on pistachio nuts, HFP: healthy animals that received free *P. acidilactici* SK cells, DIP: diabetic animals that received the immobilized *P. acidilactici* SK cells on pistachio nuts, DFP: diabetic animals that received free *P. acidilactici* SK cells. TAC: total aerobic counts, LAB: lactic acid bacteria. a *p* < 0.05 vs. HFP, b *p* < 0.05 vs. HIP, and c *p* < 0.05 vs. DFP.

As a next step, tissue-adherent microbiota were explored in four different segments of the intestine (Figure 3). In the jejunum segment, the levels of coliforms and *E. coli* were significantly higher in the diabetic rats that consumed the immobilized cells (DIP) compared to the corresponding group of healthy animals (*p* = 0.026 and *p* = 0.008, respectively, between DIP and HIP); however, similar levels were observed between the rest of the groups (*p* > 0.05). Similarly, the ileum populations of *Enterobacteriacae* (*p* = 0.002 and *p* < 0.001), coliforms (*p* = 0.007 and *p* = 0.002), *E. coli* (*p* = 0.002 and *p* < 0.001), staphylococci (*p* = 0.001 and *p* < 0.001), and bifidobacteria (*p* = 0.038 and *p* = 0.002) were higher in the diabetic animal models that consumed the free *P. acidilactici* SK cells compared to the healthy groups (HIP and HFP, respectively), but were at similar levels to the DIP group (*p* > 0.05). In the DFP group, the loads of enterococci were similar to those of the healthy groups (*p* = 1.000) and the populations of *Enterobacteriacae*, coliforms, and bifidobacteria were increased compared to those of the group of healthy animals that received the free cells (*p* < 0.001, *p* = 0.002 and *p* = 0.002, respectively). However, they were at similar levels to those of the group receiving the immobilized *P. acidilactici* SK cells on pistachio nuts (*p* = 0.433, *p* = 1.000 and *p* = 1.000). The bifidobacteria at the jejunum ranged in higher levels in the groups of diabetic animal models that received the free (*p* = 0.046 compared to HFP) and immobilized cells (*p* = 0.008 and *p* = 0.003 compared to HIP and HFP, respectively), but exhibited similar levels between the two diabetic groups (*p* = 1.000 between DIP and DFP).

Regarding the large intestine, in STZ-induced diabetic animal models (groups DIP and DFP), the levels of *Enterobacteriaceae*, coliforms, and *E. coli* were significantly increased compared to healthy animals (*p* < 0.001 for all groups), but at similar levels between the two diabetic groups (*p* = 0.508, *p* = 0.855 and *p* = 0.854, respectively) at the cecum. The counts of LAB in the DFP group were higher than in the HFP group (*p* = 0.021). Accordingly, the consumption of *L. rhamnosus* NCDC 17 and *L. rhamnosus* GG probiotic cells by T2DM rats for 6 weeks resulted in increased levels of LAB and bifidobacteria in the intestinal segment of the cecum [72]. At the colon, the level of TAC was higher in the DIP group compared to that in the HFP group (*p* = 0.041), but at similar levels compared to the other groups (*p* = 0.118 and *p* = 1.000 compared to HIP and DFP, respectively). In the STZ-induced diabetic rats that received the free *P. acidilactici* SK cells, increased populations of *Enterobacteriaceae* (*p* = 0.003 and *p* = 0.005, respectively), coliforms (*p* < 0.001 and *p* < 0.001, respectively), *E. coli* (*p* = 0.014 and *p* = 0.024, respectively), and LAB (*p* = 0.045 and *p* = 0.045, respectively) were observed compared to the groups of healthy animals (HIP and HFP), while in DIP group, the corresponding levels of these populations fluctuated at similar levels to those of the healthy groups (*p* > 0.05). In addition, the levels of coliforms in the DFP group were higher compared to those of the DIP group (*p* = 0.038). Finally, the levels of streptococci were higher in the DFP group compared to those in the HFP (*p* = 0.012) groups, but were at similar levels between the other groups (*p* > 0.05).

Studies focusing on gut microbiota generally use sole fecal samples, which mainly represent the cecal/colonic intestinal fluid content. Given that nutrient absorption takes place in the small intestine and the different conditions that are manifested throughout the GI tract, the small and large intestine are two distinct sites that consist of different microbial niches and are also different from feces. Therefore, it is crucial to map any possible ecological or physiological differences between them to better understand the immense potential of gut microbiota interactions, including probiotic-induced changes [73].

### 3.4. Microbiome Alterations Using NGS of 16S rRNA

The role of the gut microbiome in metabolic diseases has been an area of intense research over the last two decades given the rise in robust techniques that allow high-throughput sequencing at continuously more accessible costs and in short time frames. The microorganisms that are harbored in the human body perform pivotal functions that could not be otherwise accomplished. A balanced gut microbiota can promote the metabolic health of the human host, while, in a state of dysbiosis, it can contribute to the pathogenesis and manifestation of various common metabolic disorders [74], such as T1DM [68]. Microbiome profiling using 16S rRNA is currently the most widely used technology to map the differences in microbial abundances of the intestinal microbiota and was of great interest in this study. Dietary intervention for 4 weeks with presumptive functional cells of *P. acidilactici* SK led to significant differences in phylum and genus taxonomic groups, while the Shannon and Simpson diversity indices were not affected by the dietary intervention (Table S1, Supplementary Data).

At the phylum level, both factors (dietary intervention and time) significantly affected the OTUs of Actinobacteria (*p* < 0.001 and *p* = 0.001, respectively), Bacteroidetes (*p* < 0.001 for both factors), and Firmicutes (*p* = 0.002 and *p* = 0.001, respectively), and a strong (*p* < 0.001, *p* = 0.012, *p* = 0.006 for Actinobacteria, Firmicutes, and Bacteroidetes, respectively) interaction between the two factors was observed, while no significant (*p* = 0.610, *p* = 0.983, *p* = 0.513) differences in the Proteobacteria OTUs were noticed after conducting ANOVA. Given the results of the post-hoc analysis, an increased abundance of Actinobacteria was mapped in STZ-induced diabetic animals (groups DIP and DFP) and compared to that in healthy animals (HIP and HFP) (*p* < 0.001 for all groups, Table 4). An elevated presence of Actinobacteria has been reported in STZ-induced diabetic rats [19,75] and in children with T1DM [76], while in other reports, their presence was decreased compared to that in healthy people [64,77]. In this work, the most abundant phyla were Firmicutes and Bacteroidetes, as reported in other studies [78,79], and the relative percentages of these two phyla were affected by the probiotic intervention. In the HFP group, the relative abundances of Bacteroidetes and Firmicutes were at similar levels compared to baseline (*p* = 1.000 for both) after 4 weeks, while in the HIP group, after 4 weeks, there was a significant increase in the amounts of Firmicutes along with a significant decrease in the amounts of Bacteroidetes compared to baseline values (*p* = 0.005 and *p* = 0.002, respectively). This finding is in accordance with the results of a 30-day dietary intervention with pistachio nuts [19]. Furthermore, in the HFP group, the 4th week values of Bacteroidetes and Firmicutes were significantly different compared to those of the HIP group (*p* = 0.020 and *p* = 0.042, respectively). In STZ-induced diabetic animals, the relative abundances of the two phyla were similar at baseline (*p* = 1.000 for both phyla) and after 4 weeks (*p* = 1.000 and *p* = 0.839 for Firmicutes and Bacteroidetes, respectively) of supplementation with *P. acidilactici* SK cells (free or immobilized).

The levels of Firmicutes/Bacteroidetes were significantly increased in the HIP group after 4 weeks (*p* = 0.041 vs. baseline) of consumption of immobilized *P. acidilactici* SK cells on pistachio nuts. An increased Firmicutes/Bacteroidetes ratio has been associated with obesity, while a decrease has been associated with inflammatory bowel disease, both in animal models and in humans [80,81]; however, there are conflicting results in the literature. When administration of synbiotic formulation containing probiotic cells of *L. acidophilus* NCFM and *B. animalis* subsp. *lactis* Bl-04 with combinations of prebiotics was studied in a four-stage semicontinuous model system of the human colon, an increase in the Firmicutes/Bacteroidetes ratio was observed [82], in agreement with our results.

**Figure 4:**
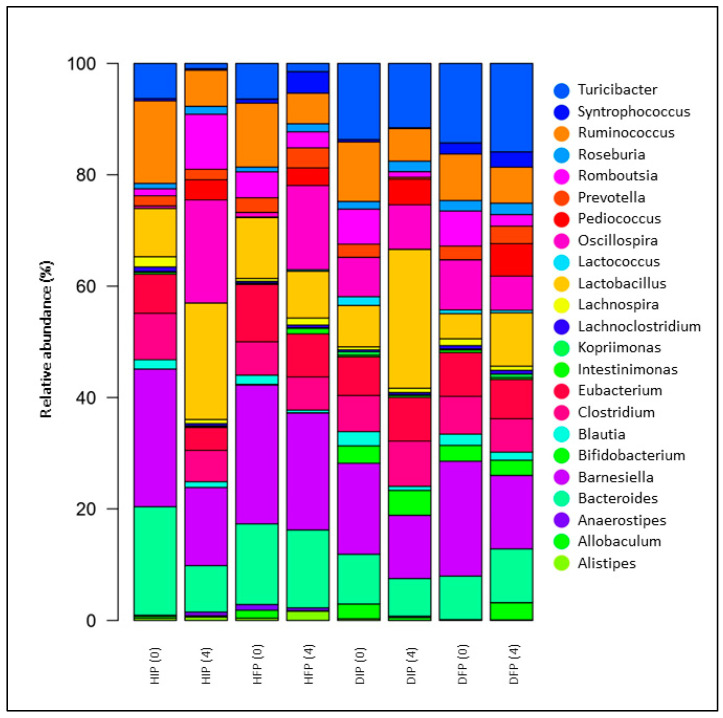
Normalized relative abundances (%) in fecal samples at genus level after 4-week administration of free or immobilized *P. acidilactici* SK cells on pistachio nuts, in healthy and STZ-induced diabetic rats, as determined by 16S rRNA NGS. HIP: healthy animals that received the immobilized *P. acidilactici* SK cells on pistachio nuts, HFP: healthy animals that received free *P. acidilactici* SK cells, DIP: diabetic animals that received the immobilized *P. acidilactici* SK cells on pistachio nuts, DFP: diabetic animals that received free *P. acidilactici* SK cells.

At the genus level, differences among the different groups and over time were also recorded (Figure 4). The consumption of free or immobilized *P. acidilactici* SK cells led to an increased presence of the genus *Pediococcus* in all groups (both in healthy and diabetic animal models) after 4 weeks (*p* = 0.003, *p* = 0.007, *p* < 0.001, and *p* < 0.001 for HIP, HFP, DIP, and DFP groups, respectively, compared to baseline values). This finding suggested that the administrated cells (both in free and immobilized form) survived the passage through the GI tract of the animal models and could be detected after NGS analysis in the stool samples, in line with several studies which reported similar results [83,84,85]. However, in a study where *B. animalis* subsp. *lactis* BB-12 cells and/or the prebiotic oligofructose were administered in diet-induced obese Sprague Dawley rats for 8 weeks, the administration of free cells did not alter the relative abundance of the genus *Bifidobacterium*. On the other hand, administration of the cells in combination with the prebiotics led to an increased presence of the genus *Bifidobacterium* [86]. Of note, one of the criteria for the selection of microorganisms to be used as probiotics is their ability to survive through GI transit and to reach their point of action (e.g., the large intestine). Nevertheless, it is possible that probiotics can manifest their beneficial actions regardless of whether this criterion is met. Thus, the survival of different genera/species or even strains must be assessed in each case.

In addition to the rise in the prevalence of the *Pediococcus* genus, there was a significant increase in the genus *Lactobacillus*. Its relative abundance was significantly increased in the groups of healthy (HIP) and diabetic animals (DIP) that were supplemented with the immobilized *P. acidilactici* SK cells on pistachio nuts, compared to baseline (*p* < 0.001 for HIP and DIP) and to the groups that received the free cells (*p* < 0.001 compared to both HFP and DFP). Increases in the prevalence of the genus *Lactobacillus* in the gut microbiome are related to beneficial effects on health, given that the prevalence of beneficial species promotes homeostasis balance in the intestinal tract, which can be disturbed in a state of dysbiosis [87], such as in T1DM.

In healthy animal models that were supplemented with *P. acidilactici* SK cells, a significant increase in the prevalence of the genus *Romboutsia* (*p* = 0.022 compared to baseline in group HIP) was also observed, as well as an increase in the genus *Oscillospira* (*p* = 0.005 in HIP and *p* = 0.022 in HFP, compared to baseline), after 4 weeks of dietary intervention. The bacterial genus *Romboutsia*, consisting of butyric acid producers, comprises gut commensals and their presence is associated with health [88,89,90], although there are reports relating an increase in the prevalence of this genus with obesity [91] or metabolic disorders [92]. An increase in the prevalence of the genus *Oscillospira* after the administration of probiotic microorganisms has been observed in other studies [93,94] and the genus is considered to include microorganisms that can be classified as next-generation probiotics [95].

In both diabetic groups (DIP and DFP), an elevated relative abundance of the *Bifidobacterium* genus was recorded compared to the healthy groups (at baseline and at the 4th week) (*p* < 0.001). However, after 4 weeks, the abundance of *Bifidobacterium* was increased (*p* < 0.001 vs. baseline) in the STZ-induced diabetic animals that received the immobilized *P. acidilactici* SK cells on pistachio nuts. Increased relative abundances of *Bifidobacterium*, a descendant of Actinobacteria, were reported in patients with T1DM [96], in Italian children with T1DM [97], and in a “TEDDY Study” that involved children before the onset of T1DM [98]. However, conflicting results showing reduced percentages have also been reported [77,99]. This result is in line with our previous study, where an increased presence of this genus was detected in diabetic rats receiving only pistachio nuts as a dietary food component for 4 weeks [19]. The administration of probiotic cells (Probio-093) to mice consuming a HFD resulted in an increased presence of the genus *Bifidobacterium* (and the phylum Actinobacteria) and beneficial effects on the metabolic health of the animals were also observed [100]. The health benefits derived from the presence of bifidobacteria are the result of a complex dynamic interaction between bifidobacteria, other microorganisms of the gut microbiota, and the human host [101]. Numerous in vitro studies have shown that bifidobacteria can inhibit pathogens through the production of organic acids [102] and antibacterial peptides [103], restoring the gut microbiome balance and preventing dysbiosis [104].

In T1DM mice, supplementation with probiotic VSL#3 altered the composition of the gut microbiome, where the presence of *Lactobacillaceae* bacteria increased, and also reduced intestinal inflammation and homeostasis in the gut immune system [67]. The administration of probiotic mixtures is suggested in the literature [91], rather than supplementation with a single strain. In the study by Wang et al. [105], the administration of 14 probiotic strains to diabetic mice (model db/db) led to an increase in the levels of Bacteroidetes, *Bifidobacterium*, *Lactobacillus*, *Roseburia*, *Prevotella*, and *Clostridium leptum*, while those of Firmicutes, Actinobacteria, *Enterococcus faecium*, *E. coli*, and *Bacteroides thetaiotaomicron* were significantly reduced compared to the control group (not given probiotics). Significant effects on the animals’ lipid profiles and plasma glucose levels were also recorded. In a clinical trial by Hou et al. [106], after the consumption of *Lacticaseibacillus casei* Zhang cells by healthy volunteers, an increased abundance of the *Lactobacillus* genus, as well as other beneficial bacteria (*Roseburia*, *Coprococcus*, and *Eubacterium rectale*) was observed, while, at the same time, the levels of opportunistic pathogens (*Blautia* and *Ralstonia*) were decreased. Certain LAB strains can suppress inflammatory responses by inhibiting various signaling pathways (such as NF-κB and MAPK) and affecting the gene expression of pro-inflammatory factors [107]. In mice models of colitis that were administered the probiotic VSL#3, although the disease was not reversed, the gut epithelial barrier function was improved, the inflammatory response was reduced, and gut microbiome balance was observed [108].

### 3.5. Stool Lactate and SCFAs

After the administration of free or immobilized *P. acidilactici* SK cells on pistachio nuts, significant effects on the profiles of SCFAs and lactic acid in feces were noticed (Table 5). ANOVA revealed that both factors (dietary intervention and time) significantly affected (*p* = 0.003 and *p* = 0.020) the acetic acid content, while a strong (*p* = 0.008) interaction between these factors on the lactic acid concentration was also observed. In contrast, no significant (*p* > 0.05) differences were noted in the rest of the SCFAs. Acetic acid was detected in the highest concentration compared to the rest of the SCFAs (*p* < 0.05) in all groups, a finding which is in agreement with the literature [30,109]. In the T1DM animal models (groups DIP and DFP), the concentration of lactic acid was significantly increased at the beginning of the study, compared to healthy animals (*p* < 0.001 compared to HIP and HFP). After 4 weeks of the dietary intervention, the lactic acid levels were reduced compared to the initial values (*p* = 0.041 and *p* = 0.004 for DIP and DFP, respectively) and ranged to similar levels to the healthy animal groups (*p* > 0.05).

Lactic acid is an intermediate molecule and can be metabolized to butyric acid, which is vital for gut health resulting in mucus synthesis [110,111,112] and maintaining the intestinal barrier. An increased presence of lactic acid is observed in inflammatory conditions [37,113] and has been reported in a similar study with rat T1DM models [114]. Probiotic consumption can lead to the elevated production of SCFAs, mainly butyrate, that could balance the intestinal cellular homeostasis by activating specific ligands that are related to the regulation of autoimmunity and, therefore, to T1DM [115]. In another study where probiotics were administered, no changes were observed in the levels of SCFAs or lactic acid [116]. A possible explanation may be that the production of metabolites by LAB takes place in the intestinal tract and so the fatty acids are likely to be used by other gut microorganisms or absorbed and transported into the systemic circulation [117].

### 3.6. Overall Findings, Limitations, and Next Steps

The fecal and intestinal tissue analysis of diabetic animals demonstrated high loads of *Enterobacteriacae*, coliforms, *E. coli*, and streptococci, a sign of the dysbiotic state occurring in T1DM. Both diabetic groups exhibited elevated levels of fecal lactic acid and inflammatory IL-1β in their plasma at baseline and at the end of the study, respectively, reflecting the inflammatory conditions that prevail in T1DM. Furthermore, in diabetic rats lower HDL-c concentrations were observed at baseline compared to the healthy groups. Since it was not clear whether these alterations were due to the presumptive probiotic strain or to the dietary intervention, further studies focusing on the mechanism of action of this strain are required to provide useful insights. After 4 weeks, in diabetic rats receiving immobilized *P. acidilactici* SK cells on pistachio nuts, lower IL-1β plasma levels compared to those receiving the dietary regimen with free cells were recorded. Both dietary regimens (immobilized or freshly adsorbed *P. acidilactici* SK cells on rats’ corn-oil-enriched food) in STZ-induced diabetic rats led to significantly increased HDL-c levels, improving their lipid profiles. Also, significant changes were induced in the intestinal microbiota and gut microbiome. After the animal models received the immobilized *P. acidilactici* SK cells on pistachio nuts, the *Enterobacteriaceae* loads were reduced in the ileum and colon, while the fecal LAB counts were increased in all groups, indicating survival of the administered strain, belonging to LAB. Moreover, the relative abundance of the genus *Pediococcus* was increased in all groups, while the genus *Lactobacillus* was elevated in the groups (both healthy and diabetic) that received the dietary regimen that included the immobilized cells. The consumption of the diet with the immobilized cells on pistachio nuts for 4 weeks resulted in the elevated presence of *Romboutsia* and *Oscillospira* genera in the healthy rats and of *Bifidobacterium* in the diabetic animals.

The results of the present study demonstrated a functional modulation of the gut microbiome, owing to the dietary regimen that included presumptive probiotic cells in immobilized form, while some alterations also occurred after administration of the dietary regimen enriched with free cells. Taking into account the changes that were mapped in the lipid profile and in the levels of inflammatory marker IL-1β of the studied animals, it is suggested that the consumption of the diet that included the immobilized cells could lead to alterations, which could be beneficial in the state of STZ-induced T1DM. A limitation of this study’s use of microbiome analysis based on the 16S rRNA gene was the use of two independent fecal samples. Although significant differences were observed, consistent with previous studies from our group [19,30,118], including a larger sample size could provide more reliable conclusions and reveal additional changes related to probiotic consumption. Another limitation of the study could be that there is, as of yet, no explanation of the specific mechanism of action of the studied cells, and these alterations need to be further explored in detail.

## 4. Conclusions

The results of the present study revealed that the consumption of immobilized *P. acidilactici* SK cells on pistachio nuts by a diabetic animal model increased the abundances of LAB and decreased the presence of opportunistic pathogens in fecal samples. Furthermore, both dietary regimens led to increased levels of HDL-c in diabetic rats after 4 weeks, while, at the end of the dietary intervention, the diabetic animals that received the immobilized *P. acidilactici* SK cells on pistachio nuts exerted lower levels of the inflammatory marker IL-1β compared to the groups that received the dietary regimen with the freshly adsorbed *P. acidilactici* SK cells on rats’ corn-oil-enriched food. However, it should be noted that these outcomes were observed in an animal model and well-designed clinical trials in humans are expected to provide helpful insights and reliable conclusions. These studies can further support the development of functional food ingredients that could be useful towards the management of T1DM.

## Figures and Tables

**Table 1:** Body weight, biochemical parameters, and insulin levels of the four groups of animals in the beginning (baseline) and at the end of the 4-week dietary intervention with free or immobilized *P. acidilactici* SK cells on pistachio nuts.

Parameter	HIP		HFP		DIP		DFP	
	Baseline	4th Week	Baseline	4th Week	Baseline	4th Week	Baseline	4th Week
Body weight (g)	422.2 ± 14.0	450.7 ± 11.8	396.6 ± 9.4	434.1 ± 8.4	381.3 ± 14.6	302.7 ± 10.3 ^a,b,c^	402.1 ± 11.9	334.6 ± 10.3 ^a,b,c^
Glucose (mg/dL)	125 ± 6	133 ± 5	100 ± 6	123 ± 10	361 ± 3 ^b,c^	407 ± 14 ^b,c^	325 ± 23 ^b,c^	393 ± 30 ^b,c^
Insulin (ng/mL)	5.1 ± 0.5	5.8 ± 0.6	4.7 ± 0.5	4.6 ± 0.8	1.3 ± 0.1 ^b,c^	1.4 ± 0.2 ^b,c^	1.2 ± 0.1 ^b,c^	1.7 ± 0.1 ^b,c^
TC (mg/dL)	116 ± 7	123 ± 8	102 ± 4	110 ± 5	89 ± 9	96 ± 6	89 ± 9	107 ± 5
TAG (mg/dL)	155 ± 21	150 ± 14	87 ± 4	91 ± 5	176 ± 19	121 ± 35	161 ± 42	139 ± 17
HDL-c (mg/dL)	94 ± 4	95 ± 6	96 ± 8	100 ± 8	52 ± 3^, c^	78 ± 4 ^a^	57 ± 5 ^b,c^	87 ± 4 ^a^
LDL-c (mg/dL)	21 ± 3	19 ± 2	26 ± 3	29 ± 3	13 ± 4	13 ± 2	12 ± 4	16 ± 2

Values are expressed as mean ± SEM (*n* = 6 per group). HIP: healthy animals that received the immobilized *P. acidilactici* SK cells on pistachio nuts, HFP: healthy animals that received free *P. acidilactici* SK cells, DIP: diabetic animals that received the immobilized *P. acidilactici* SK cells on pistachio nuts, DFP: diabetic animals that received free *P. acidilactici* SK cells. ^a^ *p* < 0.05 vs. baseline values of the same group, ^b^ *p* < 0.05 vs. HIP (of the corresponding week), and ^c^ *p* < 0.05 vs. HFP (of the corresponding week).

**Table 2:** Plasma concentrations of inflammatory markers TNF-a, IL-1β, and IL-6 of the four groups of animals at the end of the 4-week dietary intervention with free or immobilized *P. acidilactici* SK cells on pistachio nuts.

Group	TNF-a (pg/mL)	IL-1β (pg/mL)	IL-6 (pg/mL)
HIP	26.9 ± 2.6	26.3 ± 2.6	15.6 ± 1.7
HFP	26.4 ± 2.0	24.4 ± 8.0	14.0 ± 1.4
DIP	24.3 ± 3.2	57.6 ± 1.0 ^a,b,c^	14.5 ± 1.1
DFP	23.0 ± 0.7	67.9 ± 4.8 ^a,b^	16.0 ± 1.4

Values are expressed as mean ± SEM (*n* = 6 per group). HIP: healthy animals that received the immobilized *P. acidilactici* SK cells on pistachio nuts, HFP: healthy animals that received free *P. acidilactici* SK cells, DIP: diabetic animals that received the immobilized *P. acidilactici* SK cells on pistachio nuts, DFP: diabetic animals that received free *P. acidilactici* SK cells. ^a^ *p* < 0.05 vs. HIP, ^b^ *p* < 0.05 vs. HFP, and ^c^ *p* < 0.05 vs. DFP.

**Table 3:** Effect of dietary intervention with free or immobilized *P. acidilactici* SK cells on pistachio nuts on fecal microbiota populations in healthy and diabetic animals.

	HIP		HFP		DIP		DFP	
	Baseline	4th Week	Baseline	4th Week	Baseline	4th Week	Baseline	4th Week
TAC	7.70 ± 0.07	7.74 ± 0.13	7.92 ± 0.15	8.24 ± 0.12	8.25 ± 0.16	8.14 ± 0.13	8.12 ± 0.11	8.15 ± 0.04
*Enterobacteriacae*	4.37 ± 0.14	4.53 ± 0.10	4.58 ± 0.26	5.04 ± 0.14	6.00 ± 0.12 ^b,c^	5.89 ± 0.14 ^b,c^	5.70 ± 0.11 ^b,c^	5.36 ± 0.17 ^c^
coliforms	4.39 ± 0.15	4.78 ± 0.17	4.58 ± 0.23	4.99 ± 0.11	5.91 ± 0.16 ^b,c^	5.88 ± 0.14 ^b,c^	5.61 ± 0.08 ^b,c^	5.31 ± 0.19
*E. coli*	4.09 ± 0.16	4.34 ± 0.11	4.63 ± 0.19	4.94 ± 0.10	5.68 ± 0.10 ^b,c^	5.49 ± 0.12 ^b,c^	5.61 ± 0.10 ^b,c^	5.54 ± 0.11 ^c^
staphylococci	6.12 ± 0.13	5.18 ± 0.12 ^a^	6.13 ± 0.14	6.28 ± 0.13 ^b^	6.55 ± 0.12	5.71 ± 0.20 ^a^	6.47 ± 0.15	5.77 ± 0.20
enterococci	5.97 ± 0.07	5.31 ± 0.07 ^a^	5.86 ± 0.13	5.77 ± 0.19	6.17 ± 0.14	5.57 ± 0.15 ^a^	5.88 ± 0.08	5.50 ± 0.08
streptococci	7.71 ± 0.13	7.69 ± 0.14	7.94 ± 0.13	7.96 ± 0.09	8.52 ± 0.09 ^b,c^	8.18 ± 0.09	8.44 ± 0.10 ^b,c^	7.95 ± 0.16
LAB	7.32 ± 0.10	8.49 ± 0.11 ^a^	7.13 ± 0.17	8.30 ± 0.15 ^a^	7.55 ± 0.14	8.65 ± 0.07 ^a^	7.56 ± 0.16	8.57 ± 0.06 ^a^
bifidobacteria	7.58 ± 0.12	8.49 ± 0.05 ^a^	7.82 ± 0.13	8.18 ± 0.07	8.19 ± 0.21	8.65 ± 0.24	8.19 ± 0.12	8.86 ± 0.11 ^c^

Values are expressed as mean ± SEM (*n* = 6 per group). HIP: healthy animals that received the immobilized *P. acidilactici* SK cells on pistachio nuts, HFP: healthy animals that received free *P. acidilactici* SK cells, DIP: diabetic animals that received the immobilized *P. acidilactici* SK cells on pistachio, DFP: diabetic animals that received free *P. acidilactici* SK cells. TAC: total aerobic counts, LAB: Lactic Acid Bacteria. ^a^ *p* < 0.05 vs. baseline values of the same group, ^b^ *p* < 0.05 vs. HIP (of the corresponding week), and ^c^ *p* < 0.05 vs. HFP (of the corresponding week).

**Table 4:** Normalized relative abundances (%) in fecal samples at phylum level after 4-week administration of free or immobilized *P. acidilactici* SK cells on pistachio nuts, in control and STZ-induced diabetic rats, as determined by 16S rRNA NGS.

Relative Abundance (%)	HIP		HFP		DIP		DFP	
	Baseline	4th Week	Baseline	4th Week	Baseline	4th Week	Baseline	4th Week
Actinobacteria	0.0 ± 0.0	0.0 ± 0.0	0.1 ± 0.0	0.0 ± 0.0	3.1 ± 0.1 ^b,c^	4.5 ± 0.0 ^a,b,c,d^	2.9 ± 0.2 ^b,c^	2.7 ± 0.1 ^b,c^
Bacteroidetes	46.4 ± 0.9	24.8 ± 2.6 ^a^	42.4 ± 0.5	40.2 ± 0.7 ^b,d^	27.9 ± 4.7	18.4 ± 0.5	30.9 ± 1.0	26.1 ± 1.5 ^c^
Firmicutes	53.5 ± 1.0	75.0 ± 2.6 ^a^	57.4 ± 0.5	59.7 ± 0.6 ^b,d^	68.4 ± 5.3	77.0 ± 0.4	66.1 ± 1.0	70.6 ± 2.2
Proteobacteria	0.1 ± 0.1	0.2 ± 0.0	0.1 ± 0.0	0.1 ± 0.1	0.6 ± 0.6	0.1 ± 0.1	0.2 ± 0.2	0.6 ± 0.6
Firmicutes/Bacteroidetes	0.9 ± 0.0	2.1 ± 0.1 ^a^	1.4 ± 0.0	1.2 ± 0.2	1.6 ± 0.6	1.7 ± 0.7	1.2 ± 0.2	1.5 ± 0.0

Values are expressed as mean ± SEM (*n* = 2 per group). HIP: healthy animals that received the immobilized *P. acidilactici* SK cells on pistachio nuts, HFP: healthy animals that received free *P. acidilactici* SK cells, DIP: diabetic animals that received the immobilized *P. acidilactici* SK cells on pistachio nuts, DFP: diabetic animals that received free *P. acidilactici* SK cells. ^a^ *p* < 0.05 vs. baseline values of the same group, ^b^ *p* < 0.05 vs. HIP (of the corresponding week) and ^c^ *p* < 0.05 vs. HFP (of the corresponding week), and ^d^ *p* < 0.05 vs. DFP (of the corresponding week).

**Table 5:** Effect of 4-week administration of free or immobilized *P. acidilactici* SK cells on pistachio nuts on lactic acid and SCFA content (μmol/g) in the feces of healthy and STZ-induced diabetic rats.

Concentration (μmol/g)	HIP		HFP		DIP		DFP	
	Baseline	4th Week	Baseline	4th Week	Baseline	4th Week	Baseline	4th Week
Lactic acid	0.65 ± 0.08	0.59 ± 0.11	0.82 ± 0.05	0.70 ± 0.09	1.52 ± 0.09 ^b,c^	1.00 ± 0.11 ^a^	1.31 ± 0.09 ^b,c^	0.76 ± 0.08 ^a^
Acetic acid	6.91 ± 0.42	6.92 ± 0.53	6.89 ± 0.55	5.83 ± 0.88	9.74 ± 0.87	7.90 ± 0.71	9.41 ± 0.87	7.48 ± 0.64
Propionic acid	1.02 ± 0.18	1.17 ± 0.27	0.97 ± 0.11	0.89 ± 0.16	0.95 ± 0.15	0.94 ± 0.20	0.92 ± 0.10	0.96 ± 0.10
Isobutyric acid	0.05 ± 0.01	0.07 ± 0.01	0.09 ± 0.02	0.10 ± 0.02	0.07 ± 0.02	0.06 ± 0.01	0.04 ± 0.01	0.06 ± 0.01
Butyric acid	0.42 ± 0.02	0.85 ± 0.13	0.62 ± 0.07	0.92 ± 0.24	0.48 ± 0.06	0.53 ± 0.13	0.58 ± 0.03	0.74 ± 0.29
Isovaleric acid	0.03 ± 0.01	0.05 ± 0.01	0.05 ± 0.02	0.06 ± 0.02	0.02 ± 0.00	0.03 ± 0.01	0.03 ± 0.01	0.03 ± 0.01
Valeric acid	0.04 ± 0.01	0.07 ± 0.01	0.08 ± 0.02	0.09 ± 0.02	0.03 ± 0.01	0.06 ± 0.02	0.07 ± 0.01	0.04 ± 0.01

Values are expressed as mean ± SEM (*n* = 6 per group). HIP: healthy animals that received the immobilized *P. acidilactici* SK cells on pistachio nuts, HFP: healthy animals that received free *P. acidilactici* SK cells, DIP: diabetic animals that received the immobilized *P. acidilactici* SK cells on pistachio nuts, DFP: diabetic animals that received free *P. acidilactici* SK cells. ^a^ *p* < 0.05 vs. baseline values of the same group, ^b^ *p* < 0.05 vs. HIP (of the corresponding week), and ^c^ *p* < 0.05 vs. HFP (of the corresponding week).

## Data Availability

The data presented in this study are available in the main article.

## Supplementary Materials

The following supporting information can be downloaded at: https://www.mdpi.com/article/10.3390/nu16234221/s1, Table S1: Shannon’s and Simpson’s a-diversity indices in fecal samples after 4-week administration of free or immobilized on pistachio, *P. acidilactici* SK cells, in healthy and STZ-induced diabetic rats.
