# Supplementary material for: Functional Modulation of Gut Microbiota and Blood Parameters in Diabetic Rats Following Dietary Intervention with Free or Immobilized Pediococcus acidilactici SK Cells on Pistachio Nuts
Source: Nutrients. 2024 Dec 6;16(23):4221. doi: 10.3390/nu16234221 (PMC11644729; doi:10.3390/nu16234221)
Supplement: Supplementary file 1 [file nutrients-16-04221-s001.zip › nutrients-3281870-supplementary.pdf]

**Table S1.** Shannon's and Simpson's a-diversity indices in fecal samples after 4-week administration of free or immobilized *P. acidilactici* SK cells on pistachio nuts in healthy and STZ-induced diabetic rats.

| | **Shannon's** | | **Simpson's** | |
|---|---|---|---|---|
| **Groups** | baseline | $4^{th}$ week | baseline | $4^{th}$ week |
| **HIP** | 4.384±0.031 | 4.309±0.033 | 0.021±0.001 | 0.027±0.001 |
| **HFP** | 4.266±0.028 | 4.293±0.044 | 0.024±0.002 | 0.024±0.003 |
| **DIP** | 4.266±0.111 | 4.255±0.216 | 0.026±0.003 | 0.029±0.008 |
| **DFP** | 4.228±0.093 | 4.296±0.059 | 0.026±0.003 | 0.027±0.001 |

Values are expressed as mean±SEM. (n=2 per group). HIP: healthy animals that received the immobilized *P. acidilactici* SK cells on pistachio nuts, HFP: healthy animals that received free *P. acidilactici* SK cells, DIP: diabetic animals that received the immobilized *P. acidilactici* SK cells on pistachio nuts, DFP: diabetic animals that received free *P. acidilactici* SK cells.
